# Evaluation of the effectiveness and acceptability of intramuscular clozapine injection: illustrative case series

**DOI:** 10.1192/bjb.2020.6

**Published:** 2020-12

**Authors:** Rebecca Henry, Ruth Massey, Kathy Morgan, Johanne Deeks, Hannah Macfarlane, Nikki Holmes, Edward Silva

**Affiliations:** 1 Southern Health NHS Foundation Trust, UK; 2 Mersey Care NHS Foundation Trust, UK; 3 Pennine Care NHS Foundation Trust, UK; 4 Birmingham and Solihull Mental Health NHS Foundation Trust, UK; 5 Aston University, UK; 6 Nottinghamshire Healthcare NHS Foundation Trust, UK

**Keywords:** Clozapine, intramuscular, schizophrenia, treatment refusal

## Abstract

**Aims and method:**

A series of eleven patients prescribed intramuscular clozapine at five UK sites is presented. Using routinely collected clinical data, we describe the use, efficacy and safety of this treatment modality.

**Results:**

We administered 188 doses of intramuscular clozapine to eight patients. The remaining three patients accepted oral medication. With the exception of minor injection site pain and nodules, side-effects were as expected with oral clozapine, and there were no serious untoward events. Nine patients were successfully established on oral clozapine with significant improvement in their clinical presentations.

**Clinical implications:**

Although a novel formulation in the UK, we have shown that intramuscular clozapine can be used safely and effectively when the oral route is initially refused.

Clozapine remains the gold-standard intervention for treatment-resistant schizophrenia (TRS), offering a wide range of benefits.^1–5^ Case reports and series describing the use of intramuscular clozapine for patients unable or unwilling to take oral treatment have been published from authors based in Israel, the Netherlands and Australia.^6–9^ Although it is possible to successfully administer clozapine via a nasogastric tube as an alternative to the oral route, the more conventional option of an injection is usually preferable if a suitable preparation is available.^10^ Reports of the use of both intramuscular and nasogastric clozapine show that this ‘assertive’ approach can often result in improvements in mental state and a reduction in incidents and segregation, as well as facilitating progression to a less-restrictive environment. Also in practice, a stated intention to use ‘enforced’ nasogastric or intramuscular clozapine is often sufficient to persuade patients to accept the less intrusive oral route.^11,12^ Clozapine remains an underused treatment and ‘enforced’ clozapine in particular has been seen as controversial in the UK.^13–15^ The use of, and attitudes to, intramuscular clozapine in the UK have, to date, been described in poster presentations only.^16–18^ We now present the use of intramuscular clozapine in five UK settings with eleven in-patients.

## Method

Data was collected between January 2017 and July 2018 at five UK sites: two medium-secure units, two high-secure hospitals and a locked rehabilitation unit. Clinical records were used to identify patients prescribed intramuscular clozapine, and their demographics, previous, response to clozapine use, use of oral and intramuscular therapy, subsequent response as assessed by clinical team impressions, adverse effects from intramuscular treatment and subsequent stabilisation on oral therapy were recorded. Pharmacy staff were consulted to report on the nurses’ practical experience of using the intramuscular formulation.

### How to use intramuscular clozapine

#### Clozapine preparation and availability

Clozapine for injection is an unlicensed ‘special’ product made in the Netherlands by Brocacef and imported to the UK by Durbin PLC via Mawdsleys. The minimum order quantity is two packs of ten 125 mg/5 mL ampoules costing approximately £2000 in total. Hospital pharmacy departments have experienced delays with importation, supply shortages and stock being sent with a shelf life of only 2 months. When new, the ampoules have a 2-year shelf life in the dark at 25°C.

#### Legal authority

In England and Wales, incapacitous or non-consenting patients detained under the Mental Health Act (1983) may be administered drug treatments for mental disorders for longer than 3 months only if a second opinion appointed doctor approves the treatment, including the route of administration. A rationale needs to be given for the use of unlicensed preparations and so, even if the current authority includes oral and/or intramuscular antipsychotics, a separate request for this preparation will be required. The legal authority to allow enforced blood taking as part of clozapine treatment is provided by section 63 of the Mental Health Act (1983) as long as there is valid authority for the clozapine treatment itself.^19^

#### Administration and dosing

Administration is by deep intramuscular injection. Depending on the volume of injection, the gluteal, lateral thigh or deltoid injections sites were used in this series. The Brocacef preparation has no UK licence. The Netherlands licence specifies that volumes of 2–4 mL should be administered into the gluteal muscle and not the arm or thigh, and that volumes >4 mL should be given as separate injections. There is no evidence base to favour one site over another. Teams may choose to split doses exceeding 4 mL.^20^ As the bioavailability is approximately twice that of oral preparations, oral doses are halved and injections are usually given once daily. Given the practical limits of administering large volumes of intramuscular medication initial plans for titrations up to volumes of 3.5 mL (87.5 mg intramuscular clozapine is equal to 175 mg oral clozapine) may require flexibility, depending on the response. This would enable the use of intramuscular clozapine beyond the initial 14-day titration.

#### Liaison with clozapine monitoring services

Although the intramuscular preparation is an unlicensed product, the aim is to establish the patient on oral clozapine as quickly as possible, with as little intramuscular use as possible (preferably none). The treating psychiatrist, pharmacy and patient will need to be registered with a clozapine monitoring service so as to allow oral clozapine to be dispensed. In practice, patients have been registered with a monitoring service. Oral clozapine has been prescribed and, if refused, then intramuscular injections of clozapine administered. Blood monitoring at the required intervals continues, so ensuring that the patient remains registered and that oral clozapine can be dispensed. The clozapine patient-monitoring service manufacturing the relevant oral brand of clozapine has no responsibility for the use of intramuscular clozapine. Our series involved patents registered with all current UK clozapine providers.

#### Available protocols and guidelines

Several trusts have produced guidelines and suggested dosing schedules for the use of intramuscular clozapine, which are available online.^21^

## Results

### Patient characteristics

All eleven patients identified were male: ten had a primary diagnosis of schizophrenia (ICD-10 code F20)^22^ and one had a primary diagnosis of bipolar disorder (ICD-10 code F31).^22^ The indication for clozapine was treatment resistance following previous failed treatments, including high dose and antipsychotic polypharmacy. Most had demonstrated a response to clozapine treatment previously, but had discontinued owing to various patient or clinician variables: complaints about blood monitoring, sedation, and a coincidental fall in platelet count owing to immune thrombocytopenia. At least two patients had experienced severe rebound psychosis when oral clozapine was stopped. All the units included have a smoke-free policy, which had been instigated before the data collection period, and so all patients were non-smokers. See Table 1 for a summary of the patient demographics.

**Table 1:** Patient demographics

Patient	Age at first episode of psychosis (years)	Age at this admission (years)	Duration of psychosis at intramuscular clozapine prescription (years)	Previous clozapine response	Previous clozapine dose (mg/d)	Setting
1	17	24	7	Poor	1100	MSU
2	28	34	6	Partial	350	MSU
3	22	39	17	Partial	325	HSS
4	23	37	14	N/A	N/A	HSS
5	21	47	26	N/A	N/A	MSU
6	17	37	20	Yes	250	MSU
7	18	30	12	Yes	600	MSU
8	22	36	14	Partial	200	HSS
9	20	31	11	Yes	Not known	HSS
10	13	50	37	Yes	Not known	HSS
11	18	38	20	N/A	N/A	Rehab
Mean	20	37	17		471	

MSU, medium-secure unit; HSS, high-secure services; N/A, not applicable; Rehab, low-secure unit.

### Use of intramuscular clozapine

In three patients the offer to choose between the oral and intramuscular route was sufficient to establish oral clozapine maintenance treatment at between 400 and 425 mg/day, with significant benefit. In the remaining eight patients intramuscular clozapine was required, and between 1 and 99 doses were administered per patient, predominantly into the gluteal muscle, with one being given into the lateral thigh after a patient developed nodules in the gluteal muscle, and one into the deltoid muscle when the patient refused to have the clozapine by any other route (it was the first dose at only 0.25 mL, and no additional effects were noted). Seven patients resisted intramuscular administration to the extent that restraint was used on between one and nine occasions during the initial 14-day dose titration. Restraint was required to take a blood sample in two individuals, one on five occasions and the other on four occasions, and there were no adverse effects during restraint. The remaining patients did not resist. By the end of 5 months, nine patients had been established on oral clozapine, the majority of these showing improvement at doses between 150 mg and 400 mg/day (mean 228 mg/day). No serious adverse effects occurred owing to either the injection itself or associated episodes of restraint. Minor injection site pain occurred in three patients; one experienced sedation, and the patient who had 99 doses of intramuscular clozapine experienced some injection site nodules. There were no injection site abscesses or infections. The maximum reported dose of intramuscular clozapine administered was 250 mg in 10 mL, which was given across three injection sites. Tables 2 and 3 describe the use of intramuscular clozapine in this series.

**Table 2:** Use of intramuscular clozapine: titration over initial 14-day protocol (see section *How to use intramuscular clozapine*)

Patient	Route of first clozapine dose	Oral clozapine doses in titration	Intramuscular clozapine doses in titration	Restraint
1	Oral	9	5	5
2	Oral	0	14	4
3	Intramuscular	10	4	2
4	Oral	13	1	7
5	Oral	13	1	1
6	Oral	14	0	0
7	Oral	14	0	0
8	Oral	14	0	6
9	Oral	14	0	0
10	Intramuscular	0	14	2
11	Intramuscular	0	14	0
Mean		9.2	5.8	2
Total		101	53	22

**Table 3:** Use of intramuscular clozapine after 14-day initial titration

Patient	Intramuscular clozapine doses
1	0
2	14
3	5
4	10
5	0
6	0
7	0
8	21
9	0
10	85
11	0
total	135

Serum levels were obtained from two patients who had intramuscular clozapine continuously for five or more days; see Table 4. The levels were consistent with the equivalent oral doses.^23^ With regards to target plasma levels, all units aimed for the usual recommended plasma range, 0.35–0.6 mg/L, but then would be guided by individual patient symptoms and side-effects; higher than usual levels were used in certain cases.

**Table 4:** Clozapine serum levels

Intramuscular dose, mg	Equivalent oral dose, mg	Clozapine, mg/L	Norclozapine, mg/L
150	300	0.26	0.11
125	250	0.3	0.13

### Prescribing and administering experiences

Nurses were familiar with the practice of intramuscular antipsychotics and in general terms the procedure was perceived as acceptable: the injection solution was reportedly easy to draw up and, despite the bright yellow colour of the solution, the syringe markings were clearly readable and administration was easy, with very little resistance against the plunger when administering.

Nurses needed reassurance and reminding about maximum volume for single intramuscular administration, so when, for example, 10 mL was administered, three injections (4 mL + 3 mL + 3 mL) were used. Overall, nurses reported that they were confident in their ability to administer intramuscular clozapine.

Doctors required advice on bioequivalence of oral and intramuscular clozapine and how to word the prescription so that it was clear the intramuscular was only to be used if oral was declined. They also occasionally needed to be reminded to document the rationale for intramuscular treatment in clinical notes and in the associated care plan. Assistance was sometimes needed, especially initially, to complete the required application and gain approval by trust approval bodies.

In the unit where nasogastric clozapine had also previously been used, the procedure for administering intramuscular was seen as much simpler, faster and less stressful for patients. However, the limitations on dosing with the intramuscular formulation owing to volume considerations were a perceived disadvantage in patients who had experienced benefit and were poorly compliant on higher doses after the initial titration.

## Discussion

When presented with a patient with TRS who refuses clozapine and consequently faces distress, disability, risk and a potentially extended length of stay, clinicians can feel that they are dealing with an impossible problem.^5^ Alternatives to clozapine are unlikely to work and may well cause harm, although a cycle of depot changes, high dose or polypharmacy regimes may still be attempted.^24,25^ ‘Enforced’ clozapine (i.e. via nasogastric or intramuscular) is a rarity and, but for the handful of case series above, barely described in the literature; there is believed to be no previous published experience of its use in the UK. Although some NHS trusts have published guidelines for the use of intramuscular clozapine, these do not provide for all eventualities and will likely need adapting to local and individual patient circumstances, often while treatment is taking place; indeed, a flexible and pragmatic approach is more likely to succeed. Difficult decisions will include not only deciding whether to use the intramuscular route at the outset, but also when to stop treatment. Neither oral clozapine nor intramuscular clozapine will provide a solution to every patient with TRS; for example, the intramuscular route may result in patient benefit, but not within the time frame or ceiling dose allowed within a protocol agreed by the governance structure of the institution. In these cases the clinical team may need to consider higher doses and/or multiple intramuscular clozapine injections over the course of the day. A higher-strength solution for injection would be very useful and could enable intramuscular clozapine to be given as the doses increase during the titration, and to continue at higher maintenance doses if the oral dose is refused later in the treatment. Presently one unit in the study prescribes intramuscular clozapine doses lower than the equivalent oral dose if 48 h of non-adherence is approaching, essentially to prevent the need for retitration. An alternative would include the nasogastric route; however, nasogastric administration of clozapine is culturally less acceptable and less routine than intramuscular in mental health settings. With nasogastric administration, more time is needed in the restraint position, therefore reducing patient safety, and there is a risk the patient could vomit up the medication. The clinical team needs to consider whether the benefits of improvement, possibly in the long term, justify the short-term risks of daily restraint or the complications of using an unlicensed, relatively unused intramuscular drug treatment. Although in our series there were no adverse outcomes from the episodes of restraint, it does not necessarily follow that the procedure is devoid of risk. Our series is unusual compared to the experience elsewhere in that greater number of patients had a much higher number of injections. In the Israeli and Dutch series most patients were established on oral clozapine after one intramuscular dose only, almost all within 2 weeks of starting treatment with clozapine, and only one had treatment for 3 months. It is possible that our sites, which were all secure units, selected for a patient cohort that was more clinically complex and hence more difficult to treat.

In conclusion, given the lack of new treatments for TRS in recent years, it is essential that clinicians deliver the treatments that are likely to work, and clozapine in particular. The intramuscular route can be used to remove one of the impediments to its use, namely refusal of oral treatment. Although our experience was in secure settings, the use of intramuscular antipsychotic medication is used throughout different mental health settings and there is no reason to suggest that ‘enforced’ clozapine, whether using the intramuscular or any other route, should be confined to secure services. As was found in the series from the Netherlands and with nasogastric clozapine in the UK, many patients accept oral clozapine when faced with the coercive alternative, which is ultimately the desired outcome. There has been concern that the procedure itself might be aversive and painful; however, reports of injection site pain and inflammation were very low. We have demonstrated that the use of intramuscular clozapine, although not without some drawbacks, is overall easy, safe and effective.

## About the authors

**Rebecca Henry** is the lead pharmacist for education with Southern Health NHS Foundation Trust, UK. **Ruth Massey** is the lead clinical trials pharmacist and a highly specialist clinical pharmacist with Mersey Care NHS Foundation Trust, UK. **Kathy Morgan** is a senior clinical pharmacist with Pennine Care NHS Foundation Trust, UK. **Johanne Deeks** is deputy chief pharmacist in the Secure Care Pharmacy Service at Birmingham and Solihull Mental Health NHS Foundation Trust, UK. **Hannah Macfarlane** is a clinical tutor in psychiatric pharmacy in the School of Life and Health Sciences at Aston University, and the Lead Clinical Pharmacist in Secure Care at Birmingham and Solihull Mental Health NHS Foundation Trust, UK. **Nikki Holmes** is Head of Pharmacy for Forensic Services in the Pharmacy Department at Nottinghamshire Healthcare NHS Foundation Trust, UK. **Edward Silva** is a consultant forensic psychiatrist in Secure Services with Mersey Care NHS Foundation Trust, UK.
